# 

**Published:** 1898-07-02

**Authors:** 


					^ u The standard ot highest purity."?Lance# July 2nd, 1898.
CADBURY'S m o to* CADBURY'S3
COGOA ry JO. K? cocoa;
ABSOLUTELY PURE. Bn 1 NO DRUGS OR ALKALI
unspi TA /
No. 614. Vol. XXIV. Kg?] Satubday,
July 2nd, 1898.
[-SnhMriptionTenni,] pRICE TWOPENCE.

				

